# Chemical Analysis and Multi-Component Determination in Chinese Medicine Preparation Bupi Yishen Formula Using Ultra-High Performance Liquid Chromatography With Linear Ion Trap-Orbitrap Mass Spectrometry and Triple-Quadrupole Tandem Mass Spectrometry

**DOI:** 10.3389/fphar.2018.00568

**Published:** 2018-06-08

**Authors:** Jing Zhang, Wen Xu, Peng Wang, Juan Huang, Jun-qi Bai, Zhi-hai Huang, Xu-sheng Liu, Xiao-hui Qiu

**Affiliations:** Guangdong Provincial Key Laboratory of Clinical Research on Traditional Chinese Medicine Syndrome, The Second Clinical Medical College of Guangzhou University of Chinese Medicine, Guangdong Provincial Hospital of Traditional Chinese Medicine, Guangzhou, China

**Keywords:** Chinese medicine preparation, chemical analysis, multi-component determination, linear ion trap-orbitrap, quality control

## Abstract

Bupi Yishen Formula (BYF), a Chinese medicine preparation, has been clinically applied for the recovery of chronic kidney disease and for delaying its progress. Nevertheless, the chemical components in BYF have yet to be fully clarified. Ultra-high performance liquid chromatography with linear ion trap-Orbitrap mass spectrometry (UHPLC-LTQ-Orbitrap-MS^n^) and triple-quadrupole tandem mass spectrometry (UHPLC-TQ-MS/MS) methods were developed for qualitative chemical profiling and multi-components quantitative analysis in BYF. The chromatographic separation was performed on a Phenomenex Kinetex C_18_ column (2.1 × 100 mm i.d., 1.7 μm) using gradient elution of water (A) and acetonitrile (B) both containing 0.1% formic acid. Eighty-six compounds, including flavones, saponins, phenolic acids, and other compounds were authenticated or temporarily deduced according to their retention behaviors, mass mensuration, and characteristic fragment ions with those elucidated reference substances or literatures. Among the herbal medicinal materials of the formula, Astragali Radix, Codonopsis Radix, Salviae Miltiorrhizae Radix Rhizoma, and Polygoni Multiflori Radix Praeparata contributed to the bulk of the dissolved metabolites of the formula extraction. In addition, seven analytes were simultaneously determined by UHPLC-TQ-MS/MS, which was validated and has managed to determine major components in BYF. The study indicated that the established qualitative and quantitative methods would be potent and dependable analytical tools for characterizing multi-constituent in complex prescriptions decoction and provided a basis for the evaluation of bioactive components in BYF.

## Introduction

Chinese herbal medicine is the main form of clinical prevention and treatment of Traditional Chinese Medicine (TCM), the composition of which is composed of many different ingredients, and the organic combination of these different ingredients is different from adding individual ingredients simply. The material basis of Chinese herbal medicine is to coordinate and interact with each other so as to achieve the integrate function. Different from western medicine research, studies on Chinese herbal compound emphasize the integrity of the complex prescription, which should not split off from intrinsic characteristics of TCM and pursuit monomer compound (Wang et al., 2005). The material base of single herb or prescription is active substance groups. These groups of active substances are compatibly combined according to certain requirements, which act on multiple targets and thus has pleiotropic effects by multiple pathways (Xiong et al., 2015). Therefore, it is imperative to use modern advanced techniques to intrinsically explain the material basis of Chinese herbal medicine and to elaborate the connotation of compatibility and its curative effect.

Bupi Yishen Formula (BYF) is a non-herbal combination preparation of TCM which possesses the basic characterization of formula compatibility of TCM. BYF is prepared from the extract mixture of nine herbs, namely Astragali Radix, Codonopsis Radix, Atractylodis Macrocephalae Rhizoma, Poria, Dioscoreae Rhizoma, Polygoni Multiflori Radix Praeparata, Cuscutae Semen, Coicis Semen, and Salviae Miltiorrhizae Radix Rhizoma (Liu et al., 2012). The clinical application of BYF is treating and delaying the progression of chronic kidney disease, including postponing chronic renal failure symptoms, defering early and mid-renal dysfunction, delaying entering the dialysis time, and protection of residual renal function (Mao et al., 2015). Modern pharmacological studies revealed that the decoction could effectively delay glomerular filtration rate (GFR) of patients on the fourth stage of chronic kidney disease. Unambiguously, detecting and identifying the major components in BYF is a prerequisite and the hinge to disclose the active constituents and how they produce the effectiveness.

In recent years, reports on global characterizations of complicated ingredients in TCM prescriptions continues to grow steadily due to the recently rapid development of multifarious hyphenated and hybrid mass spectrometry (MS). Analytical methods have exhibited good performance in analysis of unknown targets from TCM prescriptions, containing LC-ESI/MS (Dou et al., 2009; Shaw et al., 2012), LC-TOF/MS (Sun et al., 2013), LC/MS-IT-TOF, etc. (Hao et al., 2008; Liu et al., 2016).

Ultra-high performance liquid chromatography (UHPLC) has been utilized in many bioanalytical fields in recent years due to its rapid analysis and excellent separation (Simons et al., 2009; Ha et al., 2013). Equipped with a relatively short column with a low flow rate, UHPLC usually cost a remarkably shorter analysis time to achieve the same separation efficiency as HPLC. The hybrid LTQ-Orbitrap analytical platform, being composed of an ion trap coupled with an Orbitrap mass analyzer, enables two scan types obtained at the same time. The Orbitrap provides relatively higher mass accuracy (< 3 ppm) and mass resolution than a number of other mass spectrometers, which is available for determining exact molecular formulas (Dunn et al., 2008; Tchoumtchoua et al., 2013). Moreover, multi-stage MS^n^ mass spectra can be detected using ion trap by data-dependent scan and also minimize total analysis time, owing to its trigger for fragment spectra of target ions, and avoiding duplication by dynamic exclusion settings (Qiu et al., 2013). Thus, the LTQ-Orbitrap platform provides elemental compositions as well as multiple-stage mass data, which allow fast, sensitive, and reliable detecting, thus facilitating the identification of unknown compounds. Constituents of BYF could be structurally classified based on similar carbon skeletons, which should share a similar fragmentation pathway of each type and hence generate common characteristic product ions. Thus, mass spectra analysis for structural identifications could be facilitated by proposed strategies. In our previous study, the combination of UHPLC and LTQ-Orbitrap-MS^n^ has been successfully used in analyzing multiple components in single herbal extracts (Xu et al., 2014; Wang et al., 2015; Zhang et al., 2015). In this study, we attempt to exploit it to detect and identify the TCM prescription, which contain hundreds of different chemical constituents.

The present work attempted to establish an expeditious UHPLC-LTQ-Orbitrap-MS^n^ applicable approach for rapid separation and reliable identification of major constituents in BYF extract. Several strategies were used during the process, such as diagnostic fragment ions screening and fragment monitoring. In the decoction, eighty-six components altogether were identified or tentatively identified according to retention time and MS spectra data. Besides, a quantitative analysis approach has been constructed by Ultra-high performance liquid chromatography with triple quadrupole mass spectrometry (UHPLC-TQ-MS/MS). Seven representative compounds of relatively high contents unequivocally identified, were selected as marker components to evaluate the quality of BYF. The UHPLC-LTQ-Orbitrap MS^n^, and UHPLC-TQ-MS/MS platforms were proved as potent tools for both rapid qualitative and quantitative detection and analysis of complicated constituents from natural resources and the study facilitated the comprehensive quality control of BYF.

## Experimental

### Chemicals, reagents, and materials

Chemical references including calycosin-7-O-β-D-glucopyranoside, calycosin, formononetin, astragulin, salvianolic acid B, (*E*)-2,3,5,4′-Tetrahydroxystilbene-2-O-glucopyranoside ((*E*)-THSG) Astragaloside I, Astragaloside II, Astragaloside III, Astragaloside IV, soyasaponin I, lobetyolin, emodin were bought from Must (Chengdu, China). Rosmarinic acid, lithospermic acid, formononetin-7-O-glucopyranoside were from Yuanye (Shanghai, China). Salvianolic acid A was purchased from Feiyu (Jiangsu, China). Isomucronulatol-7-O-glucoside, 9, 10-di-methoxypterocarpan-3-O-β-D-glucopyranoside were isolated from *Astragalus membranaceus* and provided by Prof. Zhu Dayuan from Shanghai Institute of Materia Medica. (*S*)-THSG, emodin-8-O-β-D-glucoside and physcion-8-O-β-D-glucoside were isolated from *Polygonum multiflorum* in our lab. The purity of each standard was determined by HPLC (≥95%) and their structures were confirmed by MS, ^1^H-NMR, and ^13^C-NMR. All references were deliquated with methanol for at a concentration of 50.0 μg/mL.

HPLC-grade Acetonitrile, methanol, and formic acid were from Sigma Aldrich (MO, USA). Ultra-pure water was prepared by a Milli-Q water system (Millipore, MA, USA). Other reagents and chemicals were of analytical grade.

Astragali Radix (No. 11050419, Neimenggu), Codonopsis Radix (No. 110653271, Gansu), Atractylodis Macrocephalae Rhizoma (No. 110600711, zhejiang), Poria (No. 110506341, Hunan), Dioscoreae Rhizoma (No. 121001014, Henan), Polygoni Multiflori Radix Praeparata (No. 110400831, Henan), Cuscutae Semen (No. 110502581, Shandong), Coicis Semen (No. 110600371, Guizhou), and Salviae Miltiorrhizae Radix Rhizoma (No. 110601741, Anhui) were from Kangmei(Guangdong, China). They were authenticated by Dr. Huang Zhihai and the specimens were preserved in Guangdong Provincial Hospital of TCM. Two batches of BYF concentrated granule was produced in the pilot-scale by Peili Pharmaceutical Co., Ltd. (NanNing, China).

### Preparation of calibration standard solutions

Standard of seven compounds was accurately weighed and dissolved in methanol separately to prepare the stock solution of each. A mixed stock solution was obtained, containing seven stock solutions, giving a concentration of 15.30 μg/mL for calycosin-7-*O*-Glc, 6.45 μg/mL for calycosin, 644.80 μg/mL for (*E*)-THSG, 6.03 μg/mL for astragulin, 15.60 μg/mL for rosmarinic acid, 8.10 μg/mL for salvianolic acid A, 0.812 μg/mL for salvianolic acid B, respectively. Daidzein (50.0 μg/mL) was also prepared with methanol to obtain the internal standard (IS) stock solution. To construct calibration curves, the mixed stock solution was continuously diluted for series concentrations at 1/2, 1/4, 1/8, 1/16, 1/32, 1/64, and 1/128 of the original one. In a 2 mL volumetric flask, 0.2 mL of each concentration solution above, as well as 100 μL IS solutions were added, and all concentrations were finally diluted to 2 mL with 18% aqueous methanol. The acquired solutions were conserved at 4°C in refrigerator until use. All the solutions were filtered through 0.22 μm membranes before analysis.

### Sample preparation

(i) Extraction of crude drugs: A total 125 g dry pieces of nine medicinal materials were mixed by prescription ratio and extracted with boiling water (1:10) for three times (45,30, and 30 min, respectively), filtered through gauze. Then three filtrates were combined and vacuum evaporated to recover the solvent at 56°C, and then BYF extract could be obtained. Extract was transferred into 250 mL volumetric flask, then adjusted to desired level with 10% methanol solution (final crude drug concentration was 0.5 g/mL). Solid-phase extraction (SPE) with C-18 column (ProElut, 200 mg, 3 mL column volume) was used for the pretreatment procedure, which had been conditioned with methanol (2 mL) and water (2 mL). After 1.0 mL of BYF extract was loaded, the column was washed by 10% methanol (2 mL), and eluted with 1.0 mL 100% methanol slowly. The dry pieces of each herb were disposed through the same procedure, thus individual decoction was obtained. All the sample solutions were passed through 0.22 μm membranes prior to analysis.

(ii) Pretreatment of decoction for quantitative study: The lyophilized powder of BYF decoction was produced by freeze-drying. 11.20 g lyophilized powder was acquired from 100 mL of BYF decoction. Five hundred and sixty milligrams of BYF was filtered, a 0.1 mL portion of which was added with 10 μL of the IS solution, and then was diluted with methanol to 5 mL. The sample solutions were passed through 0.22 μm membranes.

(iii) Pretreatment of BYF concentrated granule: Concentrated granule (2.0 g) was accurately weighed and precisely dissolved in 100 mL of 80% aqueous methanol, and then refluxed for 1 h. The extract was cooled down to room temperature, weighed and made up a deficiency by 80% methanol, which was then treated in the same way as (ii).

### UHPLC-LTQ-orbitrap-MS^n^ conditions

Chromatographic separation was conducted by a Thermo Accela UHPLC system (San Joes, USA) comprising an autosampler, a quaternary pump, a diode-array detector (DAD), and a column compartment settled to room temperature. A Phenomenex Kinetex C_18_ column (2.1 × 100 mm i.d., 1.7 μm) was utilized for sample separating. The mobile phase was mixture of water (A) and acetonitrile (B), both containing 0.1% formic acid. The elution gradient was set as follows: 0–12 min (10–25% B), 12–25 min (25–32% B), 15–42 min (32–56% B), 42–51 min (56–95% B). The injection volume of samples was 2 μL with a flow rate of mobile phase at 200 μL/min.

For qualitative experiments, a Thermo Fisher Scientific LTQ-Orbitrap XL hybrid mass spectrometer (Bremen, Germany) was hyphenated to the LC instrument via an electron spray ionization (ESI) interface. The samples were determined in negative mode. The ESI parameters were set (spray voltage was −3.5 KV; capillary temperature was 325°C; tube lens voltage was −76 V; Sheath gas and auxiliary gases were 45 and 6 units, respectively). The Orbitrap mass analyzer was set up the full scan mass range at *m/z* 120–1,200 of 30,000 resolution in centroided-type mass mode. In data-dependent MS^n^ acquisition, the most intense ions were always selected for online MS^2^-MS^3^ analysis by FT and MS^4^-MS^5^ analysis by LTQ, and dynamic exclusion detection was also conducted during the process for repetition prevention. Dynamic exclusion parameters was set as follows: Repeat count, 2; Repeat duration, 0.35 min; Exclusion duration, 1.0 min; Exclusion mass width, 3 amu. The collision energy for collision-induced dissociation (CID) was set as 30 % of maximum.

The number and types of expected atoms were fixed as follows for possible elemental composition of components: carbons ≤ 50, hydrogens ≤ 80, oxygens ≤ 30, nitrogens ≤ 2. The accuracy error threshold was set at 3 ppm. The software of Thermo Fisher Scientific Xcalibur 2.1 was applied for data analysis.

### UHPLC-TQ-MS/MS analysis

An Accela^TM^ UPLC system and a Thermo Scientific TSQ Quantum Ultra triple-quadrupole spectrometer (San Jose, USA) fitted with an ESI probe were employed for quantitative analysis. The separation column, column temperature, and the mobile phase were identical with those of qualitative conditions, with a gradient elution of 18–39% B at 0–5 min, 39–65% B at 5–7 min, 65–95% B at 7–9 min at 10–12 min with a flow rate at 250 μL/min. The injection volume was set at 5 μL.

Multiple reaction monitoring (MRM) was used for MS data acquisition and the conditions were designed as below: capillary temperature was 400°C; capillary voltage was 2.5 kV for negative mode and 3.0 kV for positive mode; sheath gas (N_2_) was pressure 40 psi; auxiliary gas was 8 psi; the dwell time was 100 ms. The detection parameters of target compounds were summarized in Table 1. Peak areas of each analyte and IS acquired in MRM mode were employed for calibration curve establishing. Data were collected and analyzed by Thermo Xcalibur 2.1.0 Software.

**Table 1:** Chromatographic retention time, MRM parameters, and collision energy for the seven investigated compounds.

**Analytes**	***t_*R*_* (min)**	**Ionization mode**	**Precursor ion (m/z)**	**Product ion (m/z)**	**Collision energy (eV)**
Calycosin-7-*O*-Glc	2.33	ESI(+)	447.13	285.10	27
(*E*)-THSG	2.65	ESI(–)	405.02	243.04	21
Astragulin	3.18	ESI(–)	447.02	255.04	35
Rosmarinic acid	3.71	ESI(–)	359.99	161.03	20
Salvianolic acid A	4.73	ESI(–)	492.99	295.03	18
Salvianolic acid B	4.21	ESI(–)	717.00	321.03	33
Calycosin	4.88	ESI(–)	283.36	268.04	24
IS daidzein	4.47	ESI(+)	254.90	199.01	23

### Validation of quantitative method

The linear calibration curves were established by the analyte/IS ratio of each analyte (peak area ratio between each analyte and IS). Diluted standard solutions were successively analyzed until a signal-to-noise ratio (S/N) 3:1 and 10:1 were reached, respectively, to measure the limit of detection (LOD) and limit of quantification (LOQ) of each target compound. The intra-day precision was evaluated by detecting six times during 1 day, while the inter-day precision was assessed for 3 days in a row. Repeatability was obtained by six independent sample solutions using identical procedure in section Sample Preparation and variations were displayed by the relative standard deviation (RSD). One sample solution was tested at room temperature at different times within 24 h for stability evaluation. The recovery test was validated by adding known amounts of mixed reference solution to sample solutions at three concentration levels.

## Results and discussion

### Optimization of extraction procedure and analysis conditions

Variable factors during extraction procedures of BYF granule, including extraction solvent (water, 50, 80, and 100% methanol), method (reflux and sonication), solvent volume (30, 60, and 100 mL), and time (15, 30, 45, and 60 min) were optimized so as to extract the compounds efficiently. The optimized method was finally determined to extract the BYF granule with 100 ml of 80% methanol by refluxed for 1 h.

**Figure 1:**
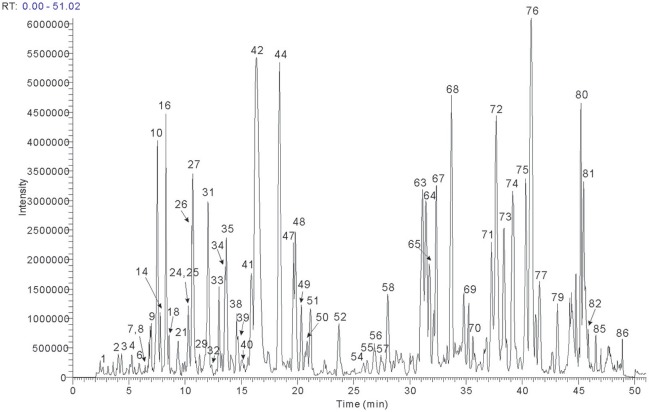
The total ion chromatogram in negtive mode of the BYF decoction.

The UHPLC conditions were optimized, containing type of column, column temperature, mobile phase system, and flow rate. The Phenomenex Kinetex C_18_ column was selected based upon our previous multi-constituents analysis. Besides, different kinds of mobile phases were tested (acetonitrile and methanol with added modifiers, including formic acid, acetic acid, and ammonium acetate). A combination of acetonitrile and water both containing 0.1% (v/v) formic acid was found not only compatible to MS analysis, but also suitable for compounds separation for qualitative analysis. Comparing the TIC of the negative and positive modes, signal response was found more sensitive to the majority of components in negative mode, thus the MS^n^ data were detected in negative mode. The total ion chromatogram of BYF was acquired for structure confirmation (shown in Figure 1).

### Characterization of constituents in BYF extract

BYF extract was analyzed using the optimized UHPLC-LTQ-Orbitrap-MS^n^ method. To elucidate the chemical components, known compounds were identified by comparing with the data of reference standards. Based on the MS^n^ analysis of the authentic compounds, the characteristic fragmentation behaviors of each type with the same carbon skeleton were conducted, and thus applied the obtained rules to structure characterization of their derivatives. For other unknown compounds, the structures were tentatively identified according to MS^n^ spectra and previous data in literatures. Eighty-six compounds in all were identified or tentatively identified (Table 2), including 15 flavones, 10 saponins, 12 phenolic acids, and other compounds. Nine herbs made markedly different chemical contributions to BYF. Specifically, the major constituents in BYF extract came from Astragali Radix (25 compounds), Codonopsis Radix (18 compounds), Salviae Miltiorrhizae (11 compounds), Cuscutae Semen (16 compounds), and Polygoni Multiflori Radix Praeparata (6 compounds).

**Table 2:** Compounds detected and identified in BYF decoction.

**No**.	***t_*R*_*/min**	**[M–H]^−^ (Mass error, ppm)**	**Characteristic fragment ions**	**Molecular formula**	**Compounds**	**Resources**
1	2.67	353.086 82 (−0.44)	MS^2^ [353]: 191.1, 179.0	C_16_H_18_O_9_	3-Caffeoyl-quinic acid	Cs
2	4.01	353.086 76 (−0.49)	MS^2^ [353]: 191.1, 179.0	C_16_H_18_O_9_	5-Caffeoyl-quinic acid	Cs
			MS^2^ [191]: 173.0, 127.0			
3	4.35	353.086 98 (−0.28)	MS^2^ [353]: 191.1, 179.0, 173.0	C_16_H_18_O_9_	4-Caffeoyl-quinic acid	Cs
			MS^2^ [191]: 173.0, 127.0			
4	5.30	469.191 35^a^ (−0.22)	MS^2^ [469]: 423.2	C_18_H_32_O_11_	(*S*)-3-Hexenyl-β-D-sophoriside	Cr
		423.185 76 (−0.32)	MS^3^ [423]: 261.1, 221.1, 179.1, 161.1			
5	5.48	350.19608^b^ (−0.12)	MS^3^ [350]: 250.1, 205.1, 161.1	C_19_H_28_NO${}_{5}^{+}$	Codonopyrrolidium A^c^	Cr
6	5.84	469.19125^a^ (−0.32)	MS^2^ [469]: 423.2	C_18_H_32_O_11_	(*E*)-2-Hexenyl-β-D-sophoriside	Cr
		423.185 91 (−0.60)	MS^3^ [423]: 261.1, 221.1, 179.1, 161.0			
7	6.44	405.118 07 (0.06)	MS^2^ [405]: 243.1	C_20_H_22_O_9_	(*Z*)-THSG	Pm
			MS^2^[243]: 225.1, 215.1, 201.0, 173.0, 149.0, 137.0			
8	6.52	677.228 09 (−0.65)	MS^2^[677]: 497.2, 453.2, 261.1	C_29_H_42_O_18_	Tangshenoside I	Cr
			MS^3^[261]: 99.1, 161.1			
9	6.96	471.207 40^a^ (0.18)	MS^2^[471]: 425.2	C_18_H_34_O_11_	Hexyl β-sophoroside	Cr
		425.201 57 (−0.17)	MS^3^[425]: 263.1, 161.0			
10	7.50	491.118 65^a^ (0.25)	MS^2^[417]: 283.1	C_22_H_22_O_10_	Calycosin-7-O-β-D-glup	Ar
			MS^3^[283]: 268.0			
			MS^4^[268]: 240.0, 224.0, 211.0, 184.0			
11	7.45	595.129 33 (−0.03)	MS^2^[595]: 463.1, 301.0, 300.0	C_26_H_28_O_16_	Quercetin 3-O-(2-O-apisyl)-galactoside	Cs
			MS^3^[300]: 271.0, 255.0			
			MS^4^[271]: 243.0, 227.01, 199.0			
12	7.70	469.133 51 (−0.54)	MS^2^ [469]: 325.1, 265.1, 235.1	C_21_H_26_O_12_	Tangshenoside IV isomer	Cr
			MS^3^ [325]: 265.1, 235.1			
13	7.74	441.196 66^a^ (0.01)	MS^2^ [395]: 263.1, 161.0	C_17_H_32_O_10_	Hexyl-(pen)-glup	Cr
		395.191 10 (−0.21)				
14	7.79	633.180 91 (−0.49)	MS^2^ [633]: 471.1, 307.1	C_30_H_34_O_15_	Unknown	Cs
			MS^3^[307]: 247.1, 205, 187, 163.1, 145.0			
15	7.97	521.128 72^a^ (0.25)	MS^2^[521]: 475.1, 359.1, 313.1, 298.1, 207.0	C_23_H_24_O_11_	Odoratin-glup	Ar
			MS^3^[313]: 298.0, 283.0, 270.0			
16	8.32	405.118 47 (−0.09)	MS^2^ [405]: 243.1	C_20_H_22_O_9_	(*E*)-THSG	Pm
			MS^2^[243]: 225.1, 215.1, 201.0, 173.0, 149.0, 137.0			
17	8.40	309.154 85^a^ (0.46)	MS^2^ [309]: 263.1	C_12_H_24_O_6_	Hexyl β-D-glup	Cr
			MS^3^ [263]: 161.1			
18	8.54	463.087 52 (0.42)	MS^2^[595]: 463.1, 301.0, 300.0	C_21_H_20_O_12_	Quercetin 3-O-galactoside (hyperoside)	Cs
			MS^3^[301]: 271.0, 255.0, 179.0, 151.0			
19	8.88	603.227 84^a^ (−0.51)	MS2 [603]: 557.2, 467.2, 341.1, 323.1	C_26_H_38_O_13_	Lobetyolinin	Cr
20	9.10	557.128 11 (−0.86)	MS^2^[557]: 405.1, 313.1, 243.1	C_27_H_25_O_13_	2,3,5,4′-Tetrahydroxystilbene-2-(galloyl)-O-glc	Pm
			MS^3^[313]: 169.0, 295.0			
21	9.32	261.133 79 (0.53)	MS^2^[261]: 187.1, 125.1	C_12_H_22_O_6_	Hexenyl-β-D-glup	Cr
22	9.40	565.191 10^a^ (−1.01)	MS^2^[519]: 357.1	C_26_H_32_O_11_	Unknown	
		519.185 85 (−0.79)	MS^2^[357]: 151.0, 136.0			
23	9.72	469.133 82 (−0.23)	MS^2^[469]: 407.1, 367.1, 325.1	C_21_H_26_O_12_	Tangshenoside V	Cr
			MS^3^[325]: 163.0, 119.1			
24	10.21	417.081 63[M-H]^−^(0.01)	MS^2^[417]: 373.1, 175.0	C_20_H_17_O_10_	Salvianolic acid D	Sm
			MS^2^[307]: 175.0			
25	10.30	537.102 29 (−1.9)	MS^2^[537]: 493.1, 339.0, 295.1	C_27_H_22_O_12_	Salvianolic acid H or I	Sm
			MS^3^[339]: 321.0, 295.1, 185.0			
26	10.60	187.097 35 (0.89)	MS^2^[187]: 125.1, 97.1	C_9_H_16_O_4_	Azelaic acid	Co / Cr
27	10.69	447.092 41 (−0.33)	MS^2^[447]: 327.0, 285.0, 284.0	C_21_H_20_O_11_	Astragulin	Cs
			MS^3^[284]: 255.0, 227.0			
28	10.83	507.113 01^a^ (−0.31)	MS^2^[507]: 461.1, 299.1	C_22_H_22_O_11_	Pratensein-7-O-Glu	Ar
			MS^3^[299]: 284.0			
			MS^4^[284]: 256.0, 227.0, 212.0			
29	11.30	477.102 60 (−1.5)	MS^2^[477]: 314.0	C_22_H_22_O_12_	Unknown	/
			MS^3^[314]: 285.0, 271.0, 243.0			
30	11.86	441.175 29^a^ (−0.23)	MS^2^ [441]: 395.1, 305.1, 215.1, 185.1, 179.1	C_20_H_28_O_8_	Lobetyolin	Cr
		395.169 65 (−0.40)				
31	12.03	359.076 51 (0.36)	MS^2^ [359]: 223.0, 197.1, 179.0, 161.0	C_18_H_6_O_8_	Rosmarimic acid	Sm
32	12.33	537.102 48 (−0.27)	MS^2^[537]: 493.1, 339.0, 295.1	C_27_H_22_O_12_	Salvianolic acid H or I	Sm
			MS^2^[493]: 321.0, 295.1			
33	13.03	723.499 08^a^ (0.78)	MS^2^[677]: 451.3	C_48_H_68_O_5_	Unknown	Cr/Am/Dr/ Ar
		677.493 90 (1.08)	MS^2^[677]: 433.4, 225.1			
34	13.56	537.102 97 (0.22)	MS^2^[537]: 493.1	C_27_H_22_O_12_	Lithospermic acid	Sm
			MS^3^[493]: 295.1			
			MS^4^[295]: 277.1, 159.0, 109.0			
35	13.69	475.123 66^a^ (0.17)	MS^2^[475]: 429.1, 267.1	C_22_H_22_O_9_	Formononetin-7-*O*-Glc	Ar
			MS^3^[267]: 252.0, 22.9, 208.1			
36	14.43	505.133 64^a^ (−0.42)	MS^2^[475]: 297.1	C_23_H_24_O_10_	6,4′-Dimethoxyisoflavone-7-*O*-Glc	Ar
			MS^3^[297]: 282.1			
			MS^4^[282]: 267.1, 254.1, 239.1			
37	14.48	823.265 87 (0.35)	MS^2^ [823]: 497.2, 453.2, 261.1	C_38_H_48_O_20_	6^‴^-Trans-p-coumaroyl-tangshenoside I	Cr
38	14.60	471.128 48 (−1.4)	MS^2^ [471]: 307.1	C_24_H_24_O_10_	Unknown	Cs
			MS^3^[307]: 247.1, 187.0, 163.1, 145.0			
39	14.74	533.128 66^a^ (−1.6)	MS^2^[533]: 487.1, 445.1, 283.1	C_24_H_24_O_11_	Calycosin-7-*O*-glc-6″-*O*-acetate	Ar
			MS^3^[283]: 268.0			
			MS^4^[268]: 240.0, 224.0, 211.0			
40	15.13	823.265 81 (0.34)	MS^2^ [823]: 497.2, 453.2, 261.1	C_38_H_48_O_20_	6^‴^-Cis-p-coumaroyl-tangshenoside I	Cr
41	15.92	269.092 59 (−0.03)	MS^2^[269]: 225.1	C_15_H_14_O_3_N_2_	Cuscutamine	Cs
			MS^3^[225]: 207.1, 183.1, 166.1, 156.1			
42	16.38	717.144 65 (-0.36)	MS^2^[717]: 519.1, 321.0	C_36_H_30_O_16_	Salvianolic acid B	Sm
			MS^3^[519]: 339.0, 321.0			
			MS^4^[321]: 293.0, 279.1, 277.1			
			MS^5^[279]: 251.1			
43	16.43	507.150 36^a^ (0.66)	MS^2^[507]: 461.1, 299.1, 284.1	C_23_H_26_O_10_	9,10-Di-methoxypterocarpan-3-*O*-β-D-glucopyranoside	Ar
			MS^3^[299]: 284.1, 269.0, 241.0			
44	18.40	283.060 73 (0.63)	MS^2^[283]: 268.0, 240.0, 224.0, 211.0	C_16_H_12_O_5_	Calycosin	Ar
45	18.54	509.165 44^a^ (0.08)	MS^2^[509]: 463.1, 445.1, 346.1, 301.1, 286.0	C_23_H_28_O_10_	Isomucronulatol-7-*O*-β-D-glucopyranoside	Ar
			MS^3^[301]: 286.1, 135.0, 109.0			
46	19.37	523.123 23 (–0.27)	MS^2^[523]: 491.1, 343.1, 325.1, 293.0	–	–	–
47	19.63	493.113 16 (−0.6)	MS^2^[493]: 295.1	C_26_H_22_O_10_	Salvianolic acid A	Sm
			MS^3^[295]: 277.1, 267, 185, 159.0, 109.0			
48	19.81	717.143 92	MS^2^[717]: 519.1	C_36_H_30_O_16_	Salvianolic acid E	Sm
		(−1.09)	MS^3^[519]: 339.0, 321,.0			
			MS^4^[321]: 279.1, 251.1			
49	20.34	551.118 35 (−1.0)	MS^2^[551]: 519.1	C_28_H_24_O_12_	Monomethyl lithospermater	Sm
			MS^3^[519]: 353.1, 321.0			
50	20.81	431.097 08(−0.19)	MS^2^[431]: 269.0	C_21_H_20_O_10_	Emodin-8-O-glc	Pm
			MS^3^[269]: 225.0, 241.0			
			MS^4^[225]: 181.0, 210.0			
51	21.14	493.112 70 (−0.22)	MS^2^[493]: 295.1	C_26_H_22_O_10_	Isomer of Salvianolic acid A	Sm
			MS^3^[295]: 277.1, 267.1, 185.1	C_36_H_30_O_15_		
52	23.68	491.097 23 (−0.04)	MS^2^[491]: 293.0, 311.1	C_26_H_20_O_10_	Salvianolic acid C	Sm
			MS^3^[293]: 276.0, 265.0, 249.1			
53	24.23	577.154 30 (−0.88)	–	C_27_H_30_O_14_	Unknown	
54	25.32	297.076 02 (0.27)	MS^2^[297]: 282.1, 267.1, 254.1, 239.1	C_17_H_14_O_5_	7-Hydroxy-6,4′-dimethoxyisoflavone	Ar
55	26.18	517.133 67^a^ (−0.38)	MS^2^[517]: 429.1, 267.1	C_24_H_24_O_10_	Formononetin-7-*O*-β-D-glycoside-6″-*O*-acetate	Ar
			MS^3^[267]: 252.0			
			MS^4^[252]: 223.1, 208.1			
56	26.85	285.039 64 (−0.27)	MS^2^[285]: 257.0, 229.1, 213.1, 169.1, 199.1, 151.0	C_15_H_10_O_6_	Kaempferol or luteolin	Cs
57	27.86	299.055 33 (0.32)	MS^2^[299]: 284.1, 271.1, 255.1, 240.1, 227.0	C_16_H_12_O_6_	Rhamnocitrin	Ar
58	28.03	327.217 10 (0.50)	MS^2^ [327]: 291.2, 229.1, 211.1, 171.1	C_18_H_32_O_5_	9,12,13-Trihydroxy-octadec-10,15-dienoic acid	Sm / Cr
			MS^3^ [229]: 211.1			
59	28.28	549.159 85^a^ (−0.42)	MS^2^[549]: 485.1, 459.1, 415.1, 299.1	C_25_H_28_O_11_	9,10-Di-methoxypterocarpan-3-*O*-β-D-glucopyranoside-acetate	Ar
			MS^3^[299]: 284.1, 269.0, 241.0			
60	29.56	991.509 34^a^ (−1.50)	MS^2^[991]: 783.5, 765.5, 489.4	C_47_H_78_O_19_	Astragaloside VI or VII	Ar
61	29.99	671.138 73 (−0.80)	MS^2^[671]: 473.1	C_35_H_29_O_14_	Unknown	-
			MS^3^[473]: 339.1, 321.0			
62	30.51	491.118 59^a^ (0.19)	MS^2^[445]: 283.1	C_22_H_22_O_10_	Physcion-8-O-glc	Pm
		445.112 92 (−0.01)	MS^2^[283]: 268.0, 240.0			
			MS^2^[240]: 212.0, 184.1			
63	31.13	267.065 92 (0.7)	MS^2^[267]: 252.0	C_16_H_12_O_4_	Formononetin	Ar
			MS^3^[252]: 223.0, 208.0, 132.0			
64	31.42	329.232 70 (0.45)	MS^2^ [329]: 311.2, 293.2, 229.1, 211.1	C_18_H_34_O_5_	9,12,13-Trihydroxy-octadec-10-enoic acid	Cr
			MS^3^ [171]: 153.0, 127.1, 125.1			
65	31.72	329.232 64 (−0.16)	MS^2^ [329]: 311.2, 293.2, 229.1, 211.1	C_18_H_34_O_5_	5,6,9-Trihydroxy-octadec-7-enoic acid	Cr / Co
			MS^3^ [229]: 211.1, 209.1			
66	33.03	991.508 61^a^ (−2.22)	MS^2^[991]: 783.5, 765.5, 621.4, 489.4	C_47_H_78_O_19_	Astragaloside V	Ar
67	32.35	843.421 45 (−0.57)	MS^2^[843]: 797.4, 779.4, 633.5	C_38_H_68_O_20_	Cuscutic acid C/Isomer	Cs
68	33.70	843.421 08 (−0.94)	MS^2^[843]: 797.4, 779.4, 633.5	C_38_H_68_O_20_	Cuscutic acid C/Isomer	Cs
69	35.23	829.456 18^a^ (−1.83)	MS^2^[783]: 651.4, 621.4, 489.4	C_41_H_68_O_14_	Astragaloside IV	Ar
		783.450 20 (−2.33)				
70	35.62	829.455 26^a^(−2.75)	MS^2^[783]: 621.4, 489.4	C_41_H_68_O_14_	Astragaloside III	Ar
		783.450 26 (−2.27)				
71	37.26	885.431 03 (−2.10)	MS^2^[885]: 839.4, 821.4, 633.3	C_40_H_70_O_21_	Unknown	Cs
72	37.70	885.431 27 (−1.31)	MS^2^[885]: 839.4, 821.4, 633.3	C_40_H_70_O_21_	Unknown	Cs
73	38.35	885.431 09 (−1.49)	MS^2^[885]: 839.4, 821.4, 633.3	C_40_H_70_O_21_	Unknown	Cs
74	39.13	885.431 27 (−1.31)	MS^2^[885]: 839.4, 821.4, 635.3	C_40_H_70_O_21_	Unknown	Cs
75	40.30	871.467 29^a^ (−1.29)	MS^2^[825]: 783.5, 765.4, 489.4	C_43_H_70_O_15_	Astragaloside II	Ar
		825.460 75 (−2.35)				
76	40.78	885.431 40 (−1.18)	MS^2^[885]: 839.4, 821.4, 633.5	C_40_H_70_O_21_	Acely-cuscutic acid C	Cs
77	41.54	681.367 55 (−1.6)	–	C_32_H_58_O_15_	Unknown	
78	42.40	987.513 61^a^ (−2.31)	MS^2^[941]: 923.5, 795.5, 615.4, 457.4, 437.4	C_48_H_78_O_18_	Soyasaponin I	Ar
		941.508 18 (−2.26)				
79	43.14	871.466 67^a^ (−1.91)	MS^2^[871]:783.5, 765.5, 717.4, 633.4, 603.4, 489.4	C_43_H_70_O_15_	Isoastragaloside II	Ar
		825.46057 (−2.52)				
80	45.19	913.477 66^a^ (-1.48)	MS^2^[867]: 825.5, 807.5, 765.5, 747.5, 729.5, 717.4, 705.5, 699.4, 633.4, 567.4, 489.4	C_45_H_72_O_16_	Astragaloside I	Ar
		867.471 07 (−2.59)				
81	45.45	295.227 36 (0.59)	MS^2^[295]: 277.2, 195.1, 171.1	C_18_H_32_O_3_	Coronaric acid	Co
			MS^3^[277]: 233.2			
82	45.49	913.476 5^a^ (−2.58)	MS^3^[867]: 825.5, 807.5, 765.5, 747.5, 729.5, 717.4, 705.4, 699.4, 633.4, 567.4, 489.4	C_45_H_72_O_16_	Isoastragaloside I	Ar
83	45.80	269.045 04 (0.59)	MS^2^[269]: 225.0	C_15_H_10_O_5_	Emodin	Pm
			MS^3^[225]: 181.0, 210.0			
84	45.86	915.493 23^a^ (−1.56)	–	C_45_H_72_O_16_	Dioscin	Dr
85	46.55	955.487 79 (−1.92)	MS^2^[955]: 891.5, 763.5, 701.4, 613.4, 523.4	C_47_H_74_O_17_	Acetylastragaloside I	Ar
86	48.91	339.231 75 (−0.3)	MS^2^[339]: 163.1	C_23_H_32_O_2_	Unknown	–

a [M+HCOO]^−^

b [M+H]^+^

c *only detected in positive mode*.

*Cs, Cuscutae Semen; Cr, Codonopsis Radix; Pm, Polygoni Multiflori Radix Praeparata; Ar, Astragali Radix; Sm, Salviae Miltiorrhizae Radix Rhizoma; Co, Coicis Semen; Am, Atractylodis Macrocephalae Rhizoma; Dr, Dioscoreae Rhizoma; P, Poria; pen, pentoside; glup, glucopyranoside*.

### Compounds from astragali radix

Isoflavones and saponins, the major bioactive compounds in Astragali Radix, have various effects such as tonic, immunostimulant, cardioprotective diuretic, and hepatoprotective properties (Xu et al., 2006; Auyeung et al., 2009). In our work, 20 compounds from RA were totally characterized in BYF, including 12 isoflavones and 8 saponins. By comparing with information of reference standards, calycosin-7-O-β-D-glycoside, ononin, calycosin, formononetin, isomucronulatol-7-O-β-D-glucoside, 9,10-diMP-3-O-glucoside, 9,10-di-methoxypterocarpan-3-O-β-D-glucopyranoside, Astragaloside I, Astragaloside II, Astragaloside III, Astragaloside IV, and soyasaponin I were identified. Based on the MS^n^ analysis of these authentic compounds, the characteristic fragmentation behaviors of isoflavones and saponins were proposed in our previous study (Zhang et al., 2015), which were applied for the structure elucidation of their derivatives.

The MS^2^ spectra of Compound 39 and Compound 55 exhibited characteristic product ions [M-C_2_H_2_O]^−^ (*m/z* 475.1 and 429.1) and [M-glu-C_2_H_2_O]^−^ (*m/z* 283.1 and 267.1), and their characteristic product ions yielded from the aglycone ion coincided with those of calycosin and formononetin. Based on the cleavage rules of loss of acetyl (42 Da) and acetylglucosyl (204 Da) groups, the two compounds were deduced as acetyl-glucoside of calycosin and formononetin.

Astragalosides from BYF decoction were mainly constituted by cycloastragenol aglycone, while aglycone ions or ions originated from the neutral loss of different glycosyl moiety in their MS^2^ spectra. Take Compound 69 as an example, the [M-H]^−^ ion was *m/z* 783.450 20 (C_41_H_67_O${}_{14}^{-}$), which easily lose the sugar units in its MS^2^ spectra and gained typical product ions at *m/z* 651, 621, 489 from the loss of one xylose ([M-132]^−^), one glucose ([M-164 (glu)]^−^), one xylose and glucose ([M-132 (xyl)-164 (glu)]^−^), respectively. In addition, one soyasaponin (Compound 78) of lower content from Astragali Radix was found in BYF decoction.

### Compounds from codonopsis radix

The identified compounds of Codonopsis Radix in BYF can be classified into four main classes, namely, phenylpropanoid glycosides, acetylene glycosides, hexyl (hexenol) glycosides (Lin et al., 2013). In (-)ESI-MS spectra of BYF decoction, apart from phenylpropanoid glycosides (including compounds 8, 12, 23, 37, and 40) existing in [M-H]^−^ ion forms, others displayed as both [M+HCOO]^−^ and [M-H]^−^ ions.

**Figure 2:**
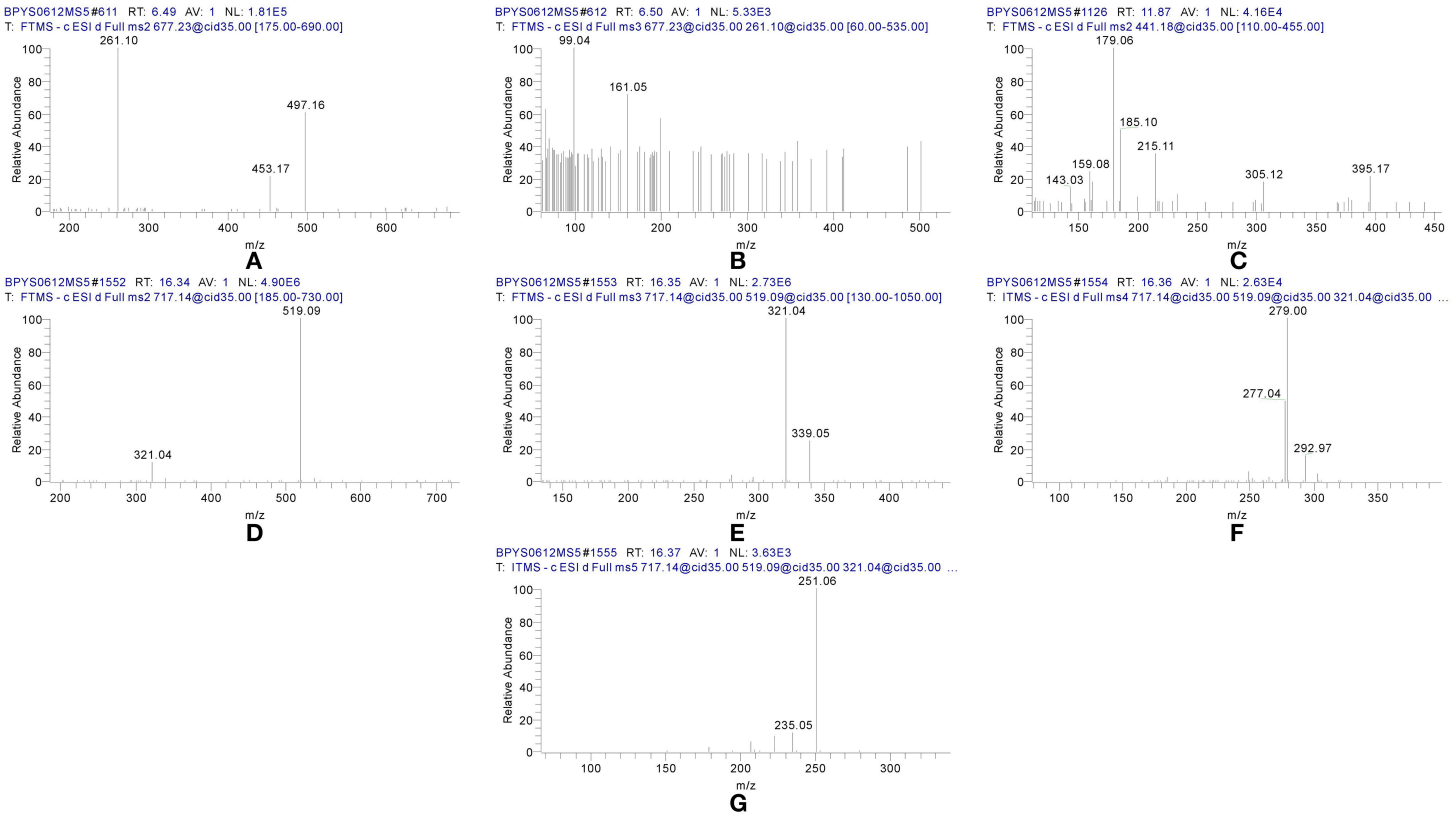
MS^n^ spectra of tangshenoside I, lobetyolin, and salvianolic acid B. **(A)** MS^2^ of 667; **(B)** MS^3^ of 261; **(C)** MS^2^ of 441; **(D)** MS^2^ of 717; **(E)** MS^3^ of 519; **(F)** MS^4^ of 321; **(G)** MS^5^ of 279.

**Figure 3:**
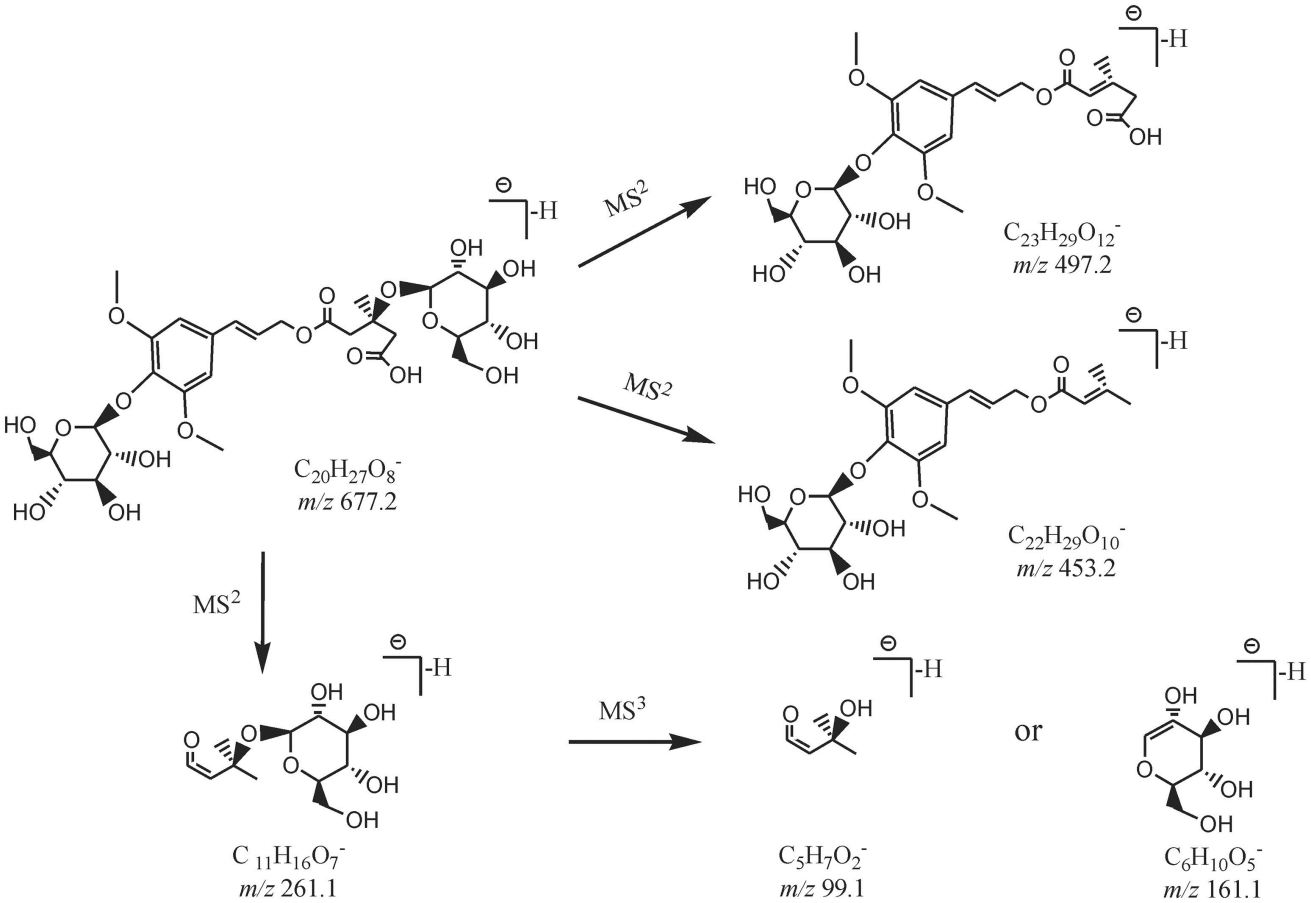
The proposed MS^n^ fragmentation pathways of tangshenoside I.

**Figure 4:**
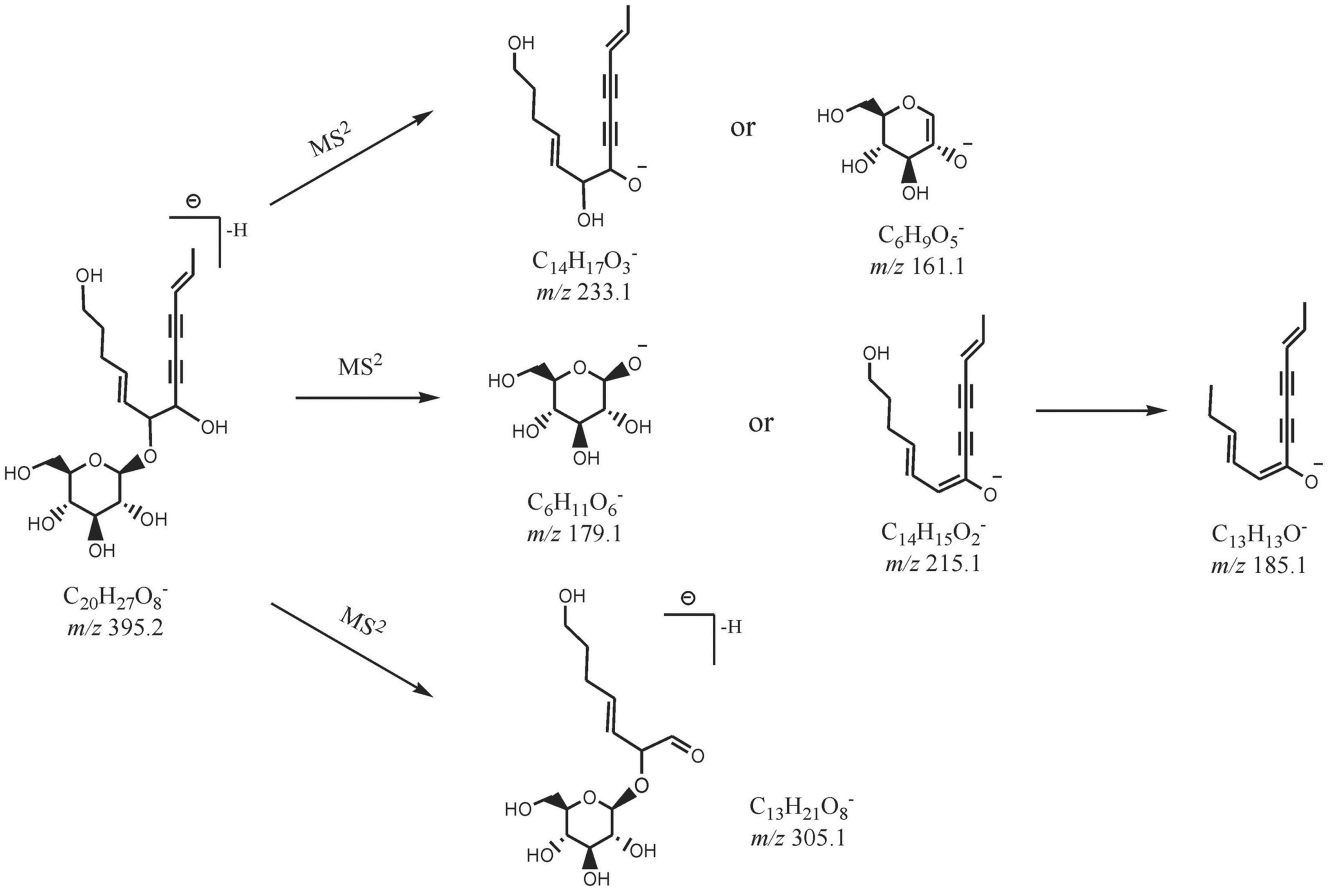
The proposed MS^n^ fragmentation pathways of lobetyolin.

By comparing the retention time values and mass data with those of the references, Compound 8 and 30 were unambiguously identified as tangshenoside I and lobetyolin, which are the representative compounds of phenylpropanoid glycosides and acetylene glycosides in Radix Codonopsis. Their MS^n^ spectra and proposed fragmentation pathways were summarized in Figures 2A–C, 3, 4, respectively. The [M-H]^−^ ion and the typical ions in the MS^2^ spectra of Compound 19 (C_26_H_3_7O${}_{13}^{-}$), namely, m/z 557.2 [M-H]^−^, 467.2 [M-C_7_H_6_]^−^, 341.1 [M-C_14_H_17_O_2_]^−^, were all 162 Da less compared to those of Compound 30, demonstrating that they have identical site cleavage. Compound 19 was therefore characterized as lobetyolinin by comparison with the literature (Kanji et al., 2003).

Compounds 37 and 40 were identified as diastereomers by the same deprotonated ions at m/z 823.265 8 (C_38_H_47_O${}_{20}^{-}$) and the same productions at m/z 497.2, m/z 453.2, and m/z 261.1, and they could be differentiated by their elution order. Their log P calculated by Discovery Studio were 0.47 and 0.59. As cis-isomers with lower polarity could by eluted relatively later than trans-isomers, compounds 37 and 40 were identified as 6^′′′^-trans- and 6^′′′^-cis-p-coumaroyl-tangshenoside I.

Compounds 9, 13, and 17 exhibited the [M+HCOO^−^]^−^ precursor ion at *m/z* 471.207 40, 441.196 66, and 309.154 85, and their MS^2^ and MS^3^ spectra all yielded ions at *m/z* 263.1 (C_12_H_23_O${}_{6}^{-}$) and 161.1 (C_6_H_9_O${}_{5}^{-}$) as the base peak, respectively. It is inferred that Compounds 9 and 13 were, respectively, substituted by an additional glucopyranoside and pentoside compared to Compound 17. Compounds 9, 13, and 17 were tentatively characterized as hexyl β-sophoroside, hexyl-(pen)-glucopyranoside, and hexyl β-D-glucopyranoside.

Isomers (Compounds 4 and 6) were obtained by the EIC of *m/z* 423. Both of Them displayed [M-H]^−^ ion at *m/z* 423.185 76 (C_18_H_31_O${}_{11}^{-}$) and their MS^2^ spectra all exhibited ion at *m/z* 261.1 [M-H-C_6_H_10_O_5_]^−^ and 161.1 (C_6_H_9_O${}_{5}^{-}$). Based on the fragmentation information and related literature (Tsai and Lin, 2010), they were primarily identified as (*S*)-3-hexenyl-β-D-sophoriside and (E)-2-hexenyl-β-D-sophoriside. Their log P calculated by Discovery Studio were −1.9 and −2.0 and cis-isomers was eluted relatively later, therefore, compounds 4 and 6 were assigned as (*S*)-3-hexenyl-β-D-sophoriside and (E)-2-hexenyl-β-D-sophoriside.

### Compounds from salviae miltiorrhizae radix rhizoma

Salviae Miltiorrhizae Radix Rhizoma was mainly composed of hydrophilic salvianolic acids and lipophilic diterpenoid quinines (Wu et al., 2007). This research adopted the water extraction method so that the major ingredients of Salviae Miltiorrhizae Radix Rhizoma in the BYF are primarily salvianolic acids. This type of compounds has high molecular weight and a lot of homologs, which display similar ESI-MS^n^ behaviors for their differentiations.

**Figure 5:**
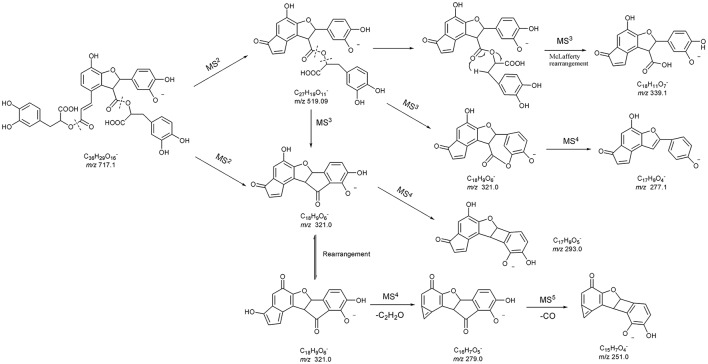
The proposed MS^n^ fragmentation pathways of salvianolic acid B.

Compound 42 displayed the [M-H]^−^ ions at *m/z* 717.144 65 with the elemental composition of C_36_H_30_O_16_. Its MS^2^ spectrum gave diagnostic fragment ions at *m/z* 519.1 and 321.0, caused by the loss of one and two molecular unit of Danshensu, respectively. In the MS^3^ spectrum, two distinctive ions at *m/z* 339.1 and 321.0, resulted from neutral loss of one danshensu and the McLafferty rearrangement, respectively, were observed. The CID of ion *m/z* 321.0 could furtherly produced MS^4^ and MS^5^ spectra. Its MS^n^ spectra, as well as fragmentation pattern were shown in Figures 2D–G, 5. As its retention time and fragmentation ions were identical with those of the reference compound, Compound 42 was identified as salvianolic acid B.

As for the fragmentation pathway of Salvianolic acids, with Danshensu as their parent nucleus, their loss of H_2_O and CO, as well as successive losses of Danshensu, occurred based on their MS spectra. Based on molecular weight and multi-stage information provided by MS^n^, combining with literatures (Hu et al., 2005; Liu et al., 2007; Zhu et al., 2010), 11 phenolic acids in the prescription were identified accurately.

### Compounds from other component herbs

Phenolic constituents, including stilbenes and anthraquinones, were regarded as the main active components in Polygoni Multiflori Radix Praeparata (Chen et al., 2012). Most of these stilbenes previously reported were mainly 2,3,5,4′-tetrahydroxy substituted type (Liu et al., 2011). Compounds 7, 16, 50, 62, and 82 were indubitably distinguished as (*E*)-THSG, (*S*)-THSG, emodin-8-O-β-D-glucoside, physcion-8-O-β-D-glucoside, and emodin, by comparing their *t*_*R*_-values and mass information with those of the standards. The MS and MS^2^ spectra of Compounds 7, 16, and 20 showed characteristic ions at *m/z* 405.118 29 and 243.065 55, representing the corresponding elemental composition of C_20_H_21_O${}_{9}^{-}$ and C_14_H_11_O${}_{4}^{-}$, which was consistent with our previous studies (Qiu et al., 2013). Fragmentation behaviors of anthraquinones were also accordance with previous results.

The components in Cuscutae Semen are mainly phenolic acids and flavonoids. For example, The MS^2^ and MS^3^ spectra of Compounds 1, 2, and 3 were in accordance with those of 3-CQA (caffeoyl-quinic acid), 5-CQA, and 4-CQA available from the literature (Zhang et al., 2007). Although the fragmentation data of 5-CQA and 4-CQA were the same, retention time of 5-CQA was shorter in reversed-phase chromatography. Elution orders of the three isomers were consistent with the reported.

Compounds 67 and 68 both exhibited [M-H]^−^ ion at *m/z* 843.421 75 (C_38_H_67_O${}_{20}^{-}$) and their MS^2^ spectra all identical ions, they were tentatively identified as cuscutic acid C and its isomers. Five isomers of acely-Cuscutic acid C (Compounds 71, 72, 73, 74, and 76) were presented by ion extraction at *m/z* 885 from TIC. They showed identical precursor ion and MS^2^ spectrum, while substituted position of their acetyl group was remained to be further studied.

### Validation of quantitative method

Seven compounds, unequivocally identified with relatively high content in both the decoction and the granule, were selected as marker components to evaluate the quality of BYF. As the extraction process that we applied was through traditional method, which is extracted by water, the major components of high content were mainly water-soluble and highly polar compounds. These compounds have been observed at the early 25 min of the UHPLC-LTQ-Orbitrap-MS spectra. Meanwhile, those less polar compounds of much lower content emerged between 25 and 50 min, However, the relatively high peak area of such compounds in mass spectra has no direct relationship to their actual content in samples. Seven compounds for quantitative analysis were mainly phenolic and flavonoid compounds. Thus daidzein, a flavonoid, was chosen as the IS due to its structural and polar similarity with the analytes, and no daidzein exist nor be detected in BYF.

Nice linearity with coefficients of determination (*R*^2^ > 0.9994) were gained for the seven compounds. LOD and LOQ tests were carried out and listed in Table 3. The intra- or inter-day variations (RSD) were within the range of 0.24–2.99, 0.64–3.04, and 0.53–2.32%, 2.16–3.72% for mixed standard solution and sample solution, respectively (Table 4). Analytes in the sample solution were found stable for 24 h with a RSD < 3.38%. Recoveries of the fourteen compounds ranged from 95.86 to 104.04% with RSD from 1.12 to 4.02% (shown in Table 5). As a result, the developed UHPLC-TQ-MS/MS method was considered as a sensitive, repeatable and accurate tool for the quantitative analysis of main compounds in BYF.

**Table 3:** Regression equations, linearity ranges, correlation coefficients, LOD, and LOQ data of the seven analytes.

**Analytes**	**Regression equations**	**Linear range (ng/mL)**	***R*^2^**	**LOD^a^ (ng/mL)**	**LOQ^b^ (ng/mL)**
Calycosin-7-*O*-Glc	*Y* = 0.00591514*X* + 0.00256763	11.48~765.0	0.9999	0.03	0.08
(*E*)-THSG	*Y* = 0.000746494*X* − 0.0040087	48.36~3,224.0	1.0000	0.02	0.04
Astragulin	*Y* = 0.000933611*X* − 0.00599332	4.523~301.5	0.9996	0.02	0.75
Rosmarinic acid	*Y* = 0.000437025*X* − 0.00906707	11.70~780.0	0.9994	0.98	3.91
Salvianolic acid A	*Y* = 0.000390993*X* − 0.0022037	6.075~405.0	0.9995	0.51	2.53
Salvianolic acid B	*Y* = 0.000286894*X* + 0.004386	609.0~40,600.0	0.9999	1.27	5.08
Calycosin	*Y* = 0.00265233*X* + 0.00139572	5.038~322.4	0.9995	0.04	0.15

a *LOD, limit of detection*.

b *LOQ, limit of quantification*.

**Table 4:** Precision, repeatability, and stability of the seven investigated compounds.

**Analytes**	**Precision (RSD %)**				**Repeatability (RSD%; *n* = 6)**	**Stability (RSD%; *n* = 6)**
	**Standard solution**		**BYF extract**			
	**Intra-day (*n* = 6)**	**Inter-day (*n* = 3)**	**Intra-day (*n* = 6)**	**Inter-day (*n* = 3)**		
Calycosin-7-*O*-Glc	2.99	2.02	0.84	2.16	1.06	1.95
(*E*)-THSG	1.48	0.64	0.77	2.03	0.45	0.36
Astragulin	2.27	2.80	0.53	3.72	1.18	2.38
Rosmarinic acid	1.07	2.74	0.73	2.90	1.24	1.11
Salvianolic acid A	0.97	2.15	2.32	4.51	3.00	1.70
Salvianolic acid B	0.24	2.74	1.02	2.83	0.74	1.42
Calycosin	2.09	3.04	0.77	3.04	0.78	3.38

**Table 5:** Recovery of the analytes.

**Analytes**	**Initial (mg)**	**Added (mg)**	**Detected (mg)**	**Recovery (%)**	**RSD (%)**
Calycosin-7-*O*-Glc	36.452	36.117	73.102	100.69	2.36
	45.465	45.147	90.417	100.76	
	54.716	54.176	110.166	103.44	
(*E*)-THSG	130.131	129.649	257.833	98.50	4.02
	161.804	161.901	328.248	102.80	
	194.186	194.367	386.662	99.03	
Astragulin	12.832	12.884	25.643	99.43	3.96
	16.183	16.100	32.122	99.00	
	19.397	19.316	37.913	95.86	
Rosmarinic acid	35.905	35.932	71.485	99.02	1.24
	45.219	44.928	89.772	99.16	
	53.911	53.924	107.457	99.30	
Salvianolic acid A	13.590	13.694	27.325	100.75	1.12
	17.258	17.107	34.256	100.74	
	20.036	20.542	40.565	101.07	
Salvianolic acid B	930.601	928.765	1, 869.590	101.10	2.11
	1, 162.726	1, 160.186	2, 309.808	98.87	
	1, 395.901	1, 393.148	2, 806.526	101.25	
Calycosin	15.531	15.505	31.663	104.04	2.58
	19.362	19.374	39.527	104.08	
	23.197	23.243	46.378	99.73	

### Application to analysis of BYF samples

**Figure 6:**
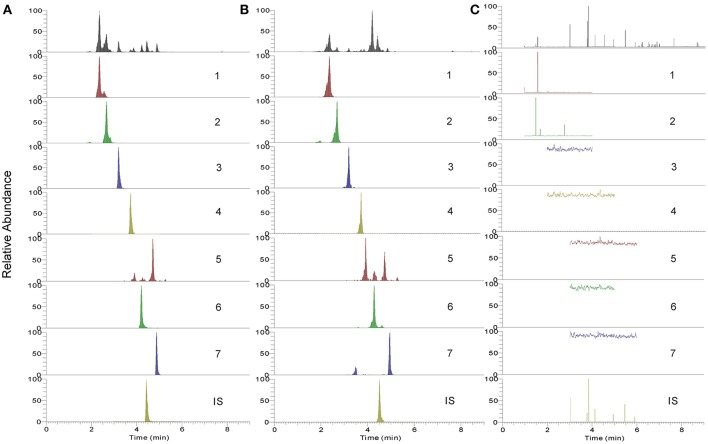
The UHPLC-TQ-MS/MS analysis MRM chromatogram of 7 analytes. **(A)** mixed standard references solution; **(B)** sample solution of BYF; **(C)** negative samples; **1**, Calycosin-7-O-Glc; **2**, (E)-THSG; **3**, astragulin; **4**, rosmarinic acid; **5**, salvianolic acid A; **6**, salvianolic acid B; **7**, Calycosin; **IS**, Daidzein.

The established UHPLC-TQ-MS/MS method was subsequently applied for quantitative analysis of both BYF decoction and its preparations. Two different batches of BYF extract powder and three different batches of BYF granule were detected using the developed method. MRM chromatograms of seven main compounds in BYF were displayed in Figure 6. The contents of the investigated compounds were determined and the outcomes were shown in Table 6.

**Table 6:** Contents (μg/g, *n* = 3) of the seven investigated compounds in the samples of BYF extract powder and BYF granule.

**Analytes**	**Contents of BYF extract powder**			**Contents of BYF granule**		
	**1**	**2**	**3**	**1**	**2**
Calycosin-7-*O*-Glc	244.124	246.061	251.827	122.741	137.846
(*E*)-THSG	382.673	466.849	438.201	270.693	305.022
Astragulin	52.899	61.430	57.947	27.192	39.693
Rosmarinic acid	10.447	29.943	23.690	63.700	103.399
Salvianolic acid A	11.303	18.126	19.853	97.021	108.978
Salvianolic acid B	5, 395.621	5, 769.462	5, 507.373	2, 059.143	2, 671.567
Calycosin	38.498	43.058	40.046	16.167	11.441

As shown in Table 6, salvianolic acid B was found as the most abundant compound, and compared with BYF decoction, the contents of most investigated compounds are relative low in concentrated granule. This difference might result from manufacturing procedures, namely, concentration, mixing, granulation, and drying processes. Meanwhile, the contents of rosmarinic acid and salvianolic acid A were much higher in concentrated granule than in BYF decoction. The variability could be explained because salvianolic acid B could be degradated and oxidized in the manufacturing procedures thus transform to other phenolic acids, such as rosmarinic acid, salvianolic acid A, lithospermic acid, etc. (Zheng and Qu, 2012).

## Conclusion

In this paper, chemical constituents of BYF were systematically investigated by UHPLC-LTQ-Orbitrap-MS^n^ and UHPLC-TQ-MS/MS methods, which provided comprehensively both qualitative and quantitative information for analysis of major components in BYF. Eighty-six compounds including flavones, saponins, phenolic acids, and other compounds were identified. The quantitative method was proved to have nice linearity, good accuracy, sensitivity, and repeatability. Although the bioactive components have not be determined, the present method will be helpful for providing the chemical basis for the further pharmacokinetic studies and effective quality evaluation of BYF, which would be of great importance for its safety use and mechanisms of action.

## Author contributions

JZ and WX performed the experiments, analyzed the data, and wrote the paper. XL, ZH, and XQ conceived and designed the experiment, contributed reagent, materials, analysis tools, and revised the manuscript. PW, JH, and JB provided constructive suggestions for this research. All authors gave approval to the final version.

### Conflict of interest statement

The authors declare that the research was conducted in the absence of any commercial or financial relationships that could be construed as a potential conflict of interest.
